# Norcantharidin in cancer therapy – a new approach to overcoming therapeutic resistance: A review

**DOI:** 10.1097/MD.0000000000037394

**Published:** 2024-03-01

**Authors:** Beilei Zeng, Xulan Chen, Lijuan Zhang, Xi Gao, Yan Gui

**Affiliations:** a Department of Oncology, Affiliated Hospital of North Sichuan Medical College, Nanchong, Sichuan, China.

**Keywords:** cancer, norcantharidin, oncology, oncotherapy, research progress, therapy resistance

## Abstract

Therapeutic resistance in cancer remains a dilemma that scientists and oncologists are eager to solve. Despite several preclinical and clinical studies dedicated to overcoming therapeutic resistance, they often do not yield the expected outcomes. This is primarily due to the multifactorial phenomenon of therapeutic resistance. Norcantharidin (NCTD) is an artificial compound derived from cantharidin that has significant anticancer efficacy without incurring serious side effects. Intriguingly, extensive research suggests that NCTD is essential for boosting anticancer efficacy and reversing treatment resistance. This review article presents a full description of how NCTD can effectively overcome cancer resistance to standard treatments such as chemotherapy, radiation, hormone therapy, and targeted therapy. We also discuss the potential prospects and challenges associated with using NCTD as a therapeutic strategy for reversing resistance to cancer therapy. We anticipate that our review will serve as a valuable reference for researchers and clinicians.

## 1. Introduction

Despite significant breakthroughs in oncotherapy, the global mortality rate of cancer remains high, with nearly 10 million deaths recorded in 2020 alone.^[1]^ Current cancer treatment strategies often fall short of their anticipated effectiveness. One of the primary reasons is therapeutic resistance, which can lead to treatment failure. Thus, elucidating the molecular mechanisms underlying therapeutic resistance and exploring new methods to overcome this obstacle are pivotal clinical aims.

Cantharidin (CTD) is the primary biologically active compound found in Cantharides, also known as Banmao in Traditional Chinese Medicine. It possesses anticancer properties and enhances the body’s immune response. However, the synthesis process is challenging and many patients experience urinary adverse effects such as hematuria and dysuria. In contrast to CTD, norcantharidin (NCTD), a demethylated analog of CTD, which exhibits enhanced bioactivity against cancer, is easier to synthesize and has fewer adverse effects, causing only mild gastrointestinal side effects without urinary adverse effects, and has been used for cancer therapy in China since the 1980s.^[2–4]^

NCTD exhibits potent anticancer properties, such as preventing cell proliferation, autophagy, migration, and metastasis, as well as the induction of apoptosis and the enhancement of anticancer immunity.^[3,4]^ The mechanisms of these properties include suppressing JAK/STAT3/TWIST signaling, the Akt pathway, the Wnt/β-catenin pathway, modulating the AMPK pathway, and the Bcl-2 protein family.^[3,4]^ Furthermore, NCTD can increase the sensitivity to antitumor drugs and reverse drug resistance in multiple human cancers, including lung cancer,^[5]^ breast cancer,^[6]^ hepatocellular cancer (HCC),^[7]^ and bladder cancer.^[8]^ Uncovering the mechanisms by which NCTD overcomes therapeutic resistance in cancer could provide additional strategies for personalized treatment. Thus, this review primarily introduces the significant role of NCTD in tumor therapeutic resistance and reviews the mechanisms of NCTD that are associated with therapeutic resistance. This review describes strategies to overcome therapeutic resistance in human cancers.

## 2. Reversing chemotherapy resistance

**Figure 1.**
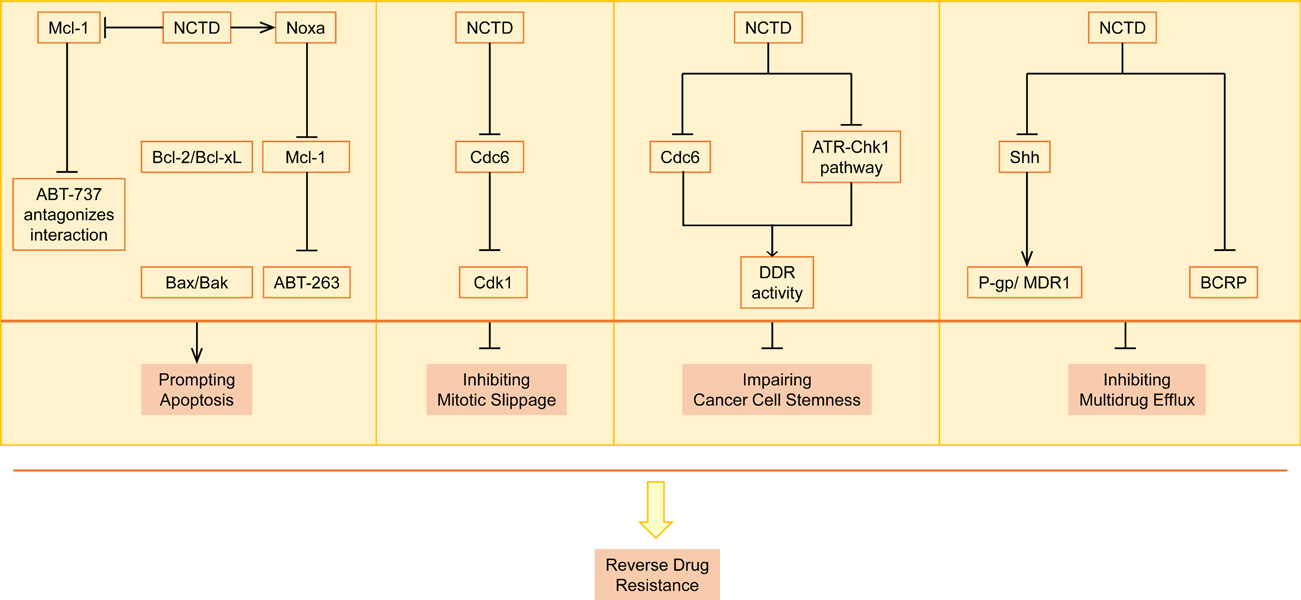
The molecular mechanisms by which NCTD overcomes chemoresistance in cancers: promoting apoptosis, inhibiting mitotic slippage, impairing cancer cell stemness, and inhibiting multidrug efflux. NCTD = norcantharidin.

Chemotherapy is a crucial and fundamental aspect of cancer treatment. Overcoming resistance to chemotherapy is a significant challenge in managing cancer. Several studies have demonstrated that NCTD can reverse chemotherapeutic resistance in cancer through various molecular mechanisms including inducing apoptosis, impairing cancer cell stemness, inhibiting multidrug efflux, and blocking mitotic slippage. The mechanisms whereby NCTD can reverse chemotherapy resistance are summarized in Figure 1 and Table 1 and discussed in more detail below.

**Table 1:** Strategies and mechanisms of reversing therapeutic resistance in cancer by NCTD.

Therapeutic strategy	Targets	Cancer type	Cell lines	Mechanisms	Experimental type	References
ABT-737	Mcl-1	HCC	HuH-7, HepG2, BEL-7402, and SMMC-7721	Suppressing Mcl-1 and causing the liberation of cytochrome c and the activation of activator caspase-9, thereby increasing the effectiveness of ABT-737	In vitro	^[7]^
ABT-263	Noxa	Neuroblastoma	SH-SY5Y and CHLA-119	Increasing Noxa expression, an innate suppressor of Mcl-1, thus leading notable improvements in ABT-263-induced anticancer activity	In vitro	^[9]^
PTX	Cdc6	HCC and cervical cancer	HepG2 and Hela	Suppressing Cdc6 expression, restoring Cdk1 activity and impeding mitotic exit, resulting in the reversal of PTX resistance	In vitro	^[10]^
Cisplatin	Cdc6 and ATR-Chk1 pathway	Bladder cancer	UMUC3 and BCSLCs	Weakening DDR activity through inhibiting Cdc6 expression and the ATR-Chk1 pathway, thereby enhancing the cytotoxicity of cisplatin to BCSLCs	In vitro and in vivo	^[8]^
DOX vinorelbine	Shh signaling and BCRP	Breast cancer	MCF-7	Suppressing Shh signaling and its downstream mdr-1/P-gp expression, and inhibiting BCRP protein expressions, thus reversing the resistance to DOX and vinorelbine	In vitro	^[11]^
Radiotherapy	Cdc6	Nasopharyngeal carcinoma and lung cancer	CNE2 and A549	Mediating the degradation of Cdc6 protein via a ubiquitin-proteasome pathway dependent on Cullin subunit 1 neddylation, thus sensitizing cancer cells to radiotherapy	In vitro and in vivo	^[12]^
tamoxifen	miR-873	Breast cancer	MCF-7, ZR75-1, T47D, and MCF-7/TamR	Regulating the miR-873-CDK3 axis and decreasing the transcriptional activity of ERα, and consequently reversing tamoxifen resistance	In vitro and in vivo	^[13]^
Sora	IL-6	HCC		Suppressing the IL-6/STAT3 signaling pathway, thereby effectively overcoming Sora resistance in HCC	In vivo	^[14]^
Everolimus	mTORC1/2	RCC	786-O and 769-P	Simultaneously inhibiting mTORC1 and mTORC2, and the downstream molecular signaling pathways, thus overcoming everolimus resistance	In vitro	^[15]^
Bortezomib	IKKα and p-IκBα	MM	U266	Suppressing the activity of IKKα and p-IκBα, leading to an increase of IκBα and subsequent inhibition of constitutive NF-κB activation, ultimately overcoming BTZ resistance	In vitro and in vivo	^[16]^
Vemurafenib	mTOR pathway	B-Raf V600E mutant melanoma	A375	Modulating of the mTOR pathway, leading to the downregulation of lipid metabolism targets, and reversing Vem resistance	In vitro	^[17]^
Gefitinib	Met	NSCLC	PC-9, HCC827, and MRC-5	Obstructing the Met/PI3K/Akt pathway, thus preventing EGFR-mutant lung cancer cells from developing resistance to EGFR-TKIs caused by endogenous and exogenous HGF	In vitro and in vivo	^[18]^

BCRP = breast cancer resistance protein, BCSLCs = bladder cancer stem cell-like cells, Cdc6 = cell division cycle 6, Cdk = cyclin-dependent kinase, DDR = DNA damage response, DOX = doxorubicin, ER = estrogen receptor, HCC = hepatocellular cancer, IKKα = I-kappa-B kinase alpha, IL = interleukin, IκBα = inhibitor of kappa B alpha, MM = multiple myeloma, mTORC = mTOR complex, NCTD = norcantharidin, NF-κB = nuclear factor κB, NSCLC = nonsmall cell lung cancer, PTX = paclitaxel, RCC = renal cell carcinoma, Shh = sonic hedgehog, Sora = sorafenib, STAT = signal transducer and activator of transcription, Vem = vemurafenib.

### 2.1. Inducing apoptosis

Apoptosis is a programmed cell death process that eliminates damaged or unwanted cells, thereby maintaining cellular homeostasis. The induction of apoptosis can enhance treatment sensitivity and overcome anticancer resistance.^[19]^

Proteins belonging to the B-cell lymphoma 2 (Bcl-2) family are important in the control of apoptotic signaling. According to their Bcl-2 homology (BH) domains, this family is functionally separated into proapoptotic (e.g., Bcl-2 associated X [Bax], Bcl-2 homologous antagonist killer [Bak], and Bcl-2 related ovarian killer [Bok]) and antiapoptotic (e.g., Bcl-2, Bcl-extra large [Bcl-xl], myeloid cell leukemia sequence 1 [Mcl-1], Bfl-1/A1, and Bcl-w) proteins.^[20]^ Dysregulated pro- and antiapoptotic proteins promote therapeutic resistance.^[21]^

ABT-737, a small-molecule BH3 mimic, is a potentially effective therapeutic agent for cancer.^[22]^ It causes apoptosis by blocking the dissociation of the proapoptotic proteins Bax and Bak, which is mediated by Bcl-2/Bcl-xL. This leads to the outer mitochondrial membrane becoming more permeable, resulting in the liberation of cytochrome c and the onset of mitochondria-mediated apoptosis in tumor cells.^[7]^ However, in a variety of malignancies, higher Mcl-1 levels promote resistance to ABT-737-induced apoptosis, presenting a significant challenge to its potential therapeutic value.^[7,22]^

Zhang et al^[7]^ demonstrated that NCTD caused the liberation of cytochrome c and the activation of activator caspase-9 in a Bax- and Bak-dependent manner, which is mainly mediated through the suppression of Mcl-1, thereby increasing the effectiveness of ABT-737-induced apoptosis in 4 hepatocellular cancer (HCC) cell lines. Their study showed that the use of NCTD in combination therapy with ABT-737 can effectively overcome resistance to ABT-737 and improve its therapeutic effectiveness in HCC.

NCTD also has the potential to enhance apoptosis triggered by another small-molecule inhibitor of Bcl-2, ABT-263, and reverse therapeutic resistance in neuroblastoma cells.^[9]^ Mcl-1 is an essential component of ABT-263 resistance in cancer cells, and Noxa, belonging to the proapoptotic subclass Bcl-2 BH3-only proteins, has been identified as an innate suppressor of Mcl-1.^[20]^ Wang et al^[9]^ revealed that the simultaneous administration of NCTD and ABT-263 led to notable improvements in ABT-263-induced apoptosis induction, inhibition of cell viability, and suppression of clonal formation, as well as an increase in Noxa expression and a decrease in Mcl-1 expression. This work conclusively demonstrated that NCTD can effectively overcome ABT-263-resistance by triggering apoptosis in neuroblastoma cells.

Collectively, these findings suggest that NCTD could potentially serve as an innovative therapeutic approach for circumventing resistance of ABT-737 and ABT-263 to chemotherapy. Given that NCTD is already used for cancer treatment in clinical settings, these findings could significantly influence the management of human HCC and neuroblastoma.

### 2.2. Blocking mitotic slippage

Paclitaxel (PTX) is a microtubule-stabilizing drug; however, its therapeutic potential is compromised by drug resistance.^[23]^ Recent studies have shown that the efficacy of antimicrotubular medicines is impeded by mitotic slippage, hence resulting in the development of therapeutic resistance. Therefore, the inhibition of mitotic slippage can overcome PTX resistance.^[23,24]^ Cell division cycle 6 (Cdc6) is a crucial regulator of the DNA replication initiation factor, and it can promote mitotic exit, that is, mitotic slippage, through the inactivation of cyclin-dependent kinase 1 (Cdk1), which is mediated by Polo-like kinase 1 (Plk1), a crucial mitotic kinase in mammals.^[25]^ He et al^[10]^ demonstrated that the occurrence of mitotic slippage was followed by an increase in the expression of Cdc6 on treatment with PTX in HepG2 and Hela cells. Therefore, depleting Cdc6 expression using NCTD or RNA interference can restore Cdk1 activity and impede mitotic exit, resulting in an increased efficacy of PTX and the reversal of PTX resistance.

### 2.3. Impairing cancer cell stemness

Cancer stem cells (CSCs), a distinct population of cells within tumors that exhibit the capability for self-renewal and the ability to differentiate into different cell types, serve an essential role in carcinogenesis. A prominent hallmark of CSCs is their inherent resistance to traditional anticancer therapies, owing to their ability to remain in a dormant state.^[26,27]^ Therefore, the focus on targeting CSCs is crucial for investigating the progression of cancer treatment and developing future therapeutic strategies. Extensive evidence has substantiated that the dysfunctional wingless-related integration site (Wnt)/β-catenin signaling pathway is essential for CSC activity.^[27,28]^ Suppressing the stemness of pancreatic cancer cells through the Wnt/β-catenin pathway using CTD or its derivative NCTD leads to enhanced efficacy of gemcitabine and erlotinib.^[29]^ In addition, studies have shown that CSCs exhibit an extremely efficient DNA damage response (DDR) system, which contributes to CSC resistance to DNA-damaging compounds like cisplatin.^[30]^ In bladder cancer stem cell-like cells (BCSLCs), NCTD can weaken DDR activity through the inhibition of chromatin-binding Cdc6 expression and the ATR-Chk1 pathway, thereby enhancing the cytotoxicity of cisplatin to BCSLCs in vitro and in vivo.^[8]^

### 2.4. Inhibiting multidrug efflux

Multidrug resistance (MDR) is an intricate phenomena, leading cancer cells to develop resistance to a diverse array of anticancer medicines without structural or molecular similarities.^[31]^ This is a substantial obstacle to the efficient treatment of malignant cancers and is the primary factor contributing to the failure of chemotherapy.^[31,32]^ The predominant mechanism underlying MDR involves the active expulsion of chemotherapeutic drugs facilitated by adenosine triphospate-binding cassette transporters. These transporters include p-glycoprotein (P-gp/multidrug resistance protein 1 [MDR1]), multidrug resistance protein 1 (MRP1), and breast cancer resistance protein (BCRP).^[31]^ Research has demonstrated that the Sonic hedgehog (Shh) pathway can facilitate the efflux of drugs in part through adenosine triphospate-binding cassette transporter, and targeting the Shh pathway may be a promising approach to tackle MDR and enhance the efficacy of chemotherapy.^[33]^ Chen et al^[11]^ demonstrated that NCTD can repress the Shh signaling pathway and the expression of its downstream mdr-1/P-gp protein. Additionally, NCTD decreases the expression of BCRP proteins, but has no impact on MPR-1 protein expression. Based on the above mechanisms, NCTD can overcome MDR to doxorubicin and vinorelbine in human MCF-7 cells.^[11]^

## 3. Reversing radioresistance

Radiotherapy is a traditional and widely utilized treatment for cancer; however, resistance to radiation limits its application and is a headache for clinicians. We have already discussed the indispensable role of Cdc6 in DDR activity and PTX-induced mitotic slippage, and Cdc6 inhibition can reverse chemotherapeutic resistance caused by mitotic slippage.^[8,10]^ Additionally, Yu et al^[34]^ have demonstrated that Cdc6 contributes to radioresistance. Therefore, targeting Cdc6 is a potential therapeutic target for overcoming radioresistance.^[34]^ Deng et al^[12]^ demonstrated that NCTD can mediate the degradation of Cdc6 protein via a ubiquitin-proteasome pathway dependent on Cullin subunit 1 neddylation, subsequently inducing cancer cell apoptosis and overcoming cancer radioresistance in radiation-adaptive CNE2 and A549 cells (Table 1). Moreover, their study presents a novel approach to tackling the problem of radiation resistance.

## 4. Overcoming endocrine therapy resistance

Tamoxifen, which is an antagonist of estrogen receptor (ER)-66, is considered as the cornerstone of endocrine therapy for the management of ER-positive breast tumors. Nonetheless, resistance to tamoxifen poses a substantial obstacle in breast cancer treatment.^[35]^ Micro-RNA (MiR)-873 is often downregulated in breast cancer, and the overexpression of MiR-873 in ER-positive breast cancer can lead to a weakening of the transcriptional activity of ERα via the regulation of ERα phosphorylation. Cyclin-dependent kinase 3 (CDK3), that has been identified as a specific target of miR-873, is overexpressed in breast cancer. A study by Cui et al^[36]^ demonstrated that miR-873 has an important function in overcoming tamoxifen resistance in breast cancer by inhibiting CDK3. Therefore, targeting miR-873/CDK3 could serve as a promising therapeutic strategy for combating tamoxifen resistance in breast cancer. Zhang et al^[13]^ demonstrated that NCTD can suppress breast cancer cell development by increasing miR-873 expression and regulating the miR-873-CDK3 axis. Consequently, the transcriptional activity of ERα is inhibited and the responsiveness of tamoxifen-resistant breast cancer cells to tamoxifen is enhanced (Table 1). Their study presented a promising therapeutic strategy for treating tamoxifen-resistant breast cancer. However, the existing research is limited, and further investigations are required to determine the efficacy of NCTD in enhancing response to tamoxifen.

## 5. Reversing targeted therapy resistance

**Figure 2.**
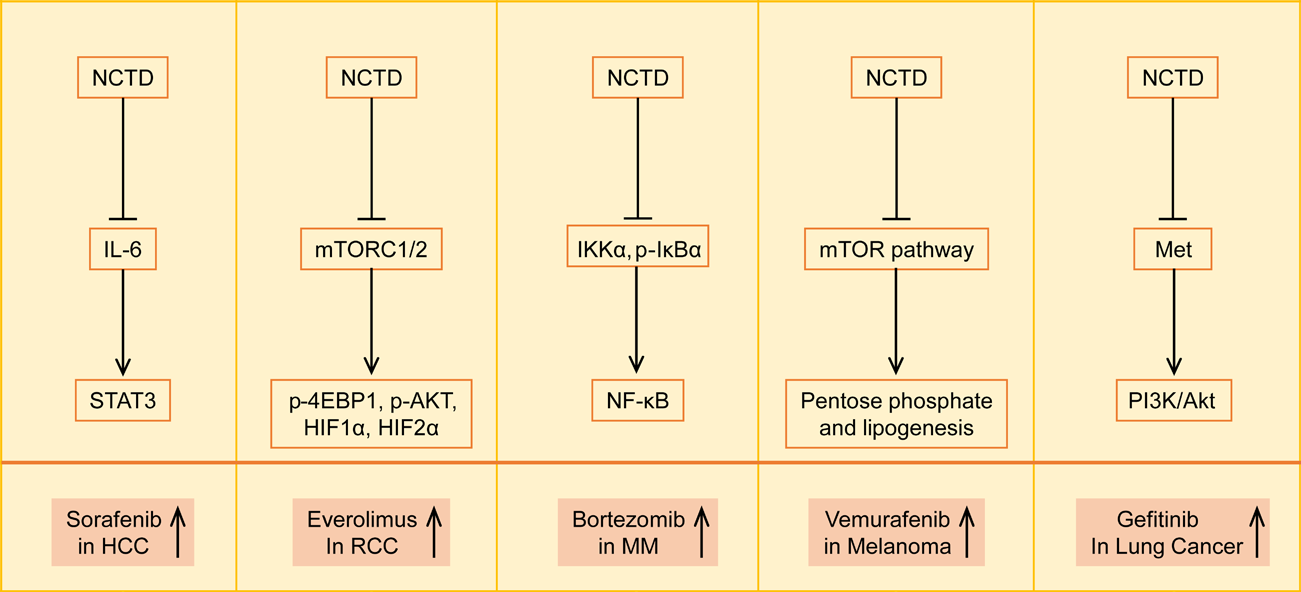
Schematic illustration of NCTD enhancing the sensitivity of targeted drugs through different molecular mechanisms. NCTD = norcantharidin.

The advent of targeted therapy has transformed the landscape of cancer treatment. However, targeted therapies inevitably result in drug resistance, which poses a challenge for oncologists. Emerging evidence suggests that NCTD can overcome resistance to targeted drugs (Fig. 2 and Table 1).

### 5.1. Sorafenib

In 2007, the US Food and Drug Administration (FDA) granted approval for sorafenib (Sora), a multikinase inhibitor that prevents angiogenesis and cancer cell proliferation, as an initial therapeutic option for advanced unresectable HCC.^[37]^ The median time to radiologic advancement is 5.5 months,^[38]^ primarily due to drug resistance, which limits the efficacy of Sora therapy. Gaining insight into the molecular mechanisms underlying Sora resistance and enhancing treatment efficacy is the initial step toward enhancing the prognosis of HCC. Interleukin-6 (IL-6), which is a proinflammatory cytokine with potent hepatocyte mitogenic properties, binding to IL-6 receptor (IL-6Rα) and initiating downstream signaling pathways, facilitates hepatic regeneration and accelerates HCC progression.^[39]^ Elevated levels of IL-6 enhance several signaling pathways, including IL-6/STAT3, leading to the development of drug-resistant HCC cells.^[40]^ Emerging evidence indicates that targeting IL-6/STAT3 can reverse Sora resistance.^[41,42]^ Yousef et al^[14]^ demonstrated that NCTD can enhance Sora-induced antitumor immunity, inhibit cancer cell viability, and attenuate tumor angiogenesis and metastasis by suppressing the IL-6/STAT3 signaling pathway, thereby potentiating the susceptibility of HCC to Sora and overcoming Sora resistance in HCC. Their study demonstrated that the combined use of NCTD and Sora could effectively address the issue of Sora resistance in HCC.

### 5.2. Everolimus

Everolimus, a mammalian target of rapamycin (mTOR) complex (mTORC)1 inhibitor, has been identified as a potentially effective alternative therapy in patients with metastatic renal cell carcinoma (RCC), who have received multiple previous lines of therapy.^[15,43]^ Nevertheless, the development of resistance to everolimus is unavoidable. Acquired resistance to everolimus can occur through the disruption of negative feedback loops that are often triggered by the activation of mTORC1/S6K/EIF4EBP1. The feedback loops may hinder the activation of mTOR complex 2 (mTORC2) and its corresponding substrate, v-Akt murine thymoma viral oncogene (AKT). When the mTORC1/S6K/EIF4EBP1 pathway is blocked, the negative feedback mechanism is disrupted. This results in the activation of AKT through mTORC2, which in turn leads to an increase in the levels of hypoxia-inducible transcription factor (HIF) and everolimus resistance.^[43,44]^ Therefore, the use of dual mTORC1/2 inhibitors may provide a more efficacious therapeutic alternative.^[15]^ The administration of NCTD can inhibit the signaling pathways of both mTORC1 and mTORC2, as well as the downstream molecular signaling pathways including phospho-(*p*)-eukaryotic translation initiation factor 4E-binding protein 1 (4EBP1), p-AKT, HIF1α, and HIF2α. Thus, combining NCTD with everolimus presents a promising treatment approach for RCC with everolimus resistance by simultaneously inhibiting mTORC1 and mTORC2.^[15]^ Further clinical research is needed to determine how to screen patients for everolimus resistance. This would enable NCTD to be used as part of the initial therapy to avoid the occurrence of drug resistance. In vivo and clinical studies are needed to explore the best combination of methods, mode of administration, and safety before mTORC1/2 inhibitors can be applied in clinical practice.

### 5.3. Bortezomib

Bortezomib (BTZ), an inhibitor of the 26S proteasome, effectively inhibits proteasomal degradation and has received approval for administration in patients diagnosed with both newly occurring and relapsed cases of multiple myeloma (MM).^[45]^ Nevertheless, approximately 30% of individuals exhibit an inadequate response to BTZ treatment. Additionally, the incidence of peripheral neuritis resulting from BTZ administration ranges from 37% to 44% among patients diagnosed with MM, which, along with the inadequate response, limits its use.^[16,46]^ Thus, a combination drug therapy may offer a reliable strategy to overcome drug resistance and reduce single-drug toxicity. Nuclear factor κB (NF-κB) constitutive activation, which is related to both MM cell activity and BTZ resistance, hinges on the activity and degradation of inhibitor of kappa B alpha (IκBα).^[47]^ NCTD can augment the antimyeloma effects of BTZ and impede the growth of MM cells by suppressing the activity of I-kappa-B kinase alpha (IKKα) and phosphorylation of IκBα, leading to an increase of IκBα and subsequent inhibition of constitutive NF-κB activation. This presents a new opportunity to overcome BTZ resistance, suggesting that the concurrent use of NCTD and BTZ could reduce the required BTZ dose, ultimately enhancing therapeutic effectiveness and decreasing the occurrence of peripheral neuritis.^[16]^

### 5.4. Vemurafenib

*BRAF* mutations are found in 40% to 60% of cutaneous melanomas, and the replacement of valine with glutamic acid at codon 600 (B-Raf^V600E^) accounts for approximately 90% of these mutations.^[48]^ The small-molecule kinase inhibitor vemurafenib (Vem) exhibits a selective targeting mechanism, specifically inhibiting the active form of B-Raf^V600E^, and was approved by the FDA in 2011.^[48]^ However, acquired resistance presents a major barrier to its clinical use. Melanoma cells with the B-Raf^V600E^ mutation exhibit distinct metabolic alterations compared with their nonmutated parental counterparts. The metabolic changes seen in cancer cells induce the emergence of novel dependencies on specific metabolites, hence facilitating treatment resistance.^[49]^ The increase of the pentose phosphate pathway and lipogenesis caused by Vem resistance might potentially be mitigated by NCTD via modulation of the mTOR pathway, leading to the downregulation of lipid metabolism targets. Hence, NCTD exhibits potential as a viable option for manipulating the metabolic processes of cancer cells and addresses the challenges posed by resistance to Vem in B-Raf^V600E^ mutant melanoma.^[17]^

### 5.5. Gefitinib

Activating mutations in the epidermal growth factor receptor (*EGFR*) gene are found in approximately 10% to 15% of individuals of European descent and 40% to 60% of Asian individuals diagnosed with nonsmall cell lung cancer (NSCLC).^[50]^ Gefitinib, the first EGFR tyrosine kinase inhibitor (EGFR-TKI) to obtain FDA approval in 2003, has been used as a primary therapeutic option for these specific individuals.^[51]^ However, the emergence of acquired resistance to EGFR-TKIs in lung cancer expressing EGFR-mutant proteins may be attributed to the excessive expression of hepatocyte growth factor (HGF), which activates the Met receptor, triggering the downstream phosphoinositide 3-kinase (PI3K)/Akt pathway. Additionally, prolonged exposure to HGF expedites the development of clones that are resistant to EGFR-TKIs.^[52]^ When coupled with gefitinib, NCTD may prevent EGFR-mutant lung cancer cells from developing resistance to EGFR-TKIs caused by endogenous and exogenous HGF by blocking the phosphorylation of Met, and subsequently obstructing the PI3K/Akt pathway. This has been demonstrated using an in vivo experimental model, suggesting that NCTD is a potential agent for the development of preventive interventions targeting EGFR-TKI-acquired resistance in NSCLC.^[18]^

## 6. Conclusions and future perspectives

Despite numerous breakthroughs in anticancer therapy, the frequent emergence of therapeutic resistance remains a significant clinical challenge, often leading to treatment failure in patients with cancer. Therapeutic resistance arises from a complex combination of various factors, encompassing the biological attributes of the tumor itself, the tumor microenvironment, and the selective pressure of tumor treatment.^[53]^ Finding efficient ways to overcome anticancer resistance with minimal side effects is a challenging task for oncologists. NCTD, a synthetic analog of CTD, retains its antitumor activity with unique characteristics, including small-molecule size, simple synthesis procedure, low levels of toxicity and leukocytosis effect, across a variety of tumors.^[2,54]^

In this review, we have summarized the mechanisms whereby NCTD reverses drug resistance in preclinical studies, signaling the commencement of a new era in the field of anticancer treatment resistance. Furthermore, oncologists and scientists have invested significant efforts developing bifunctional targeted drugs by combining NCTD with other anticancer medicines. Zhao et al^[55]^ have successfully designed and synthesized a bifunctional targeted drug composed of 3 components: camptothecin, alanine, and NCTD (CPT-Ala-Nor conjugates). The CPT-Ala-Nor conjugates have displayed robust efficacy against a variety of cancer cell lines in vitro, in addition to displaying favorable binding affinity to crucial mitotic components, such as Top I and CDC25B.^[55]^ Although use of these conjugates or combining NCTD with other existing antitumor treatment strategies show promise in reversing antitumor therapeutic resistance in cancers, several questions remain unanswered. Ascertaining whether the role of NCTD in reversing drug resistance can be generally applied to the majority of cancers and drugs is challenging. Furthermore, this review focuses on preclinical studies because clinical case studies are scarce. Moreover, determining the clinically safe dosages, timing, and strategies for this combination therapy require further evaluation. Lastly, further clinical studies are needed to determine the populations that would derive specific benefits from NCTD. In essence, the combined application of anticancer therapy and NCTD is still in its infancy and a long journey of exploration is required before clinical application can be realized.

## Acknowledgments

We thank Editage (www.editage.cn) for English language editing.

## Author contributions

**Conceptualization:** Beilei Zeng.

**Investigation:** Beilei Zeng, Xulan Chen.

**Methodology:** Lijuan Zhang.

**Writing– original draft:** Beilei Zeng.

**Writing– review & editing:** Xi Gao, Yan Gui.
